# Recombinant human thrombopoietin promotes hematopoietic reconstruction after severe whole body irradiation

**DOI:** 10.1038/srep12993

**Published:** 2015-09-25

**Authors:** Chao Wang, Bowen Zhang, Sihan Wang, Jing Zhang, Yiming Liu, Jingxue Wang, Zeng Fan, Yang Lv, Xiuyuan Zhang, Lijuan He, Lin Chen, Huanzhang Xia, Yanhua Li, Xuetao Pei

**Affiliations:** 1 Stem Cell and Regenerative Medicine Lab, Beijing Institute of Transfusion Medicine, Beijing 100850, China; 2 South China Research Center for Stem Cell & Regenerative Medicine, AMMS, Guangzhou 510005, China; 3 School of Life Science and Bio Pharmaceutics, Shenyang Pharmaceutical University, Shenyang 110016, China

## Abstract

Recombinant human thrombopoietin (rHuTPO) is a drug that is used clinically to promote megakaryocyte and platelet generation. Here, we report the mitigative effect of rHuTPO (administered after exposure) against severe whole body irradiation in mice. Injection of rHuTPO for 14 consecutive days following exposure significantly improved the survival rate of lethally irradiated mice. RHuTPO treatment notably increased bone marrow cell density and LSK cell numbers in the mice after sub-lethal irradiation primarily by promoting residual HSC proliferation. In lethally irradiated mice with hematopoietic cell transplantation, rHuTPO treatment increased the survival rate and enhanced hematopoietic cell engraftment compared with the placebo treatment. Our observations indicate that recombinant human TPO might have a therapeutic role in promoting hematopoietic reconstitution and HSC engraftment.

Accidental ionizing radiation exposure induces vital organ dysfunction syndromes in healthy individuals in radiological scenarios. The hematopoietic system is a radiosensitive organ that is highly susceptible to damage^1^,^2^,^3^, and such damage can result in death. Clinically, radiation therapy or chemotherapy for patients with malignant diseases often leads to serious hematopoietic system damage. Modulation of the hematopoietic stem cell (HSC) population is considered to be key for realizing long-term survival of patients. To date, effective therapeutic drugs targeting the hematopoietic stem cell regeneration have not been approved for clinical use. However, several strategies are being used to mitigate radiation damage to the hematopoietic system. Several studies of cytokines have confirmed the enhancement of hematopoietic recovery following myelosuppression^4^,^5^,^6^,^7^. IL-3, IL-6, IL-11 and SCF can stimulate multilineage hematopoietic recovery after otherwise lethal total body irradiation^8^,^9^,^10^,^11^,^12^. G-CSF or GM-CSF is routinely used in conjunction with radiation therapy to alleviate the symptoms of neutropenia^13^,^14^,^15^. Erythropoietin is a cytokine that stimulates erythropoiesis and can improve the symptoms of anemia in patients. Recombinant human thrombopoietin (rHuTPO) is another cytokine that is an important drug in the treatment of thrombocytopenia that acts by activating the TPO receptor MPL^16^. In addition to these growth factors that are used clinically and exert lineage-dominant responses, some potential candidates have also been reported to be capable of mitigating radiation injury to the hematopoietic system in animal models^17^,^18^,^19^,^20^. The identification of target-specific treatments that can stimulate the regeneration of multilineage repopulating cells is still an unmet challenge.

TPO is an important regulator and clinical drug for promoting megakaryopoiesis and platelet production^21^,^22^. The interaction of TPO with its receptor MPL exhibits important roles in hematopoiesis, HSC self-renewal and quiescence^23^,^24^,^25^,^26^. Both TPO and MPL knockout mice display reduced numbers of HSCs in the bone marrow, significantly fewer megakaryocytes and platelets^27^,^28^, and exogenous TPO transiently increases the proportions of quiescent HSCs in the niche^29^. These data further support the role of TPO in hematopoietic recovery^30^. Given the clinical use of rHuTPO (rHuTPO 335, TPIAO) in the treatment of thrombopenia^31^,^32^,^33^, further research on its role in hematopoietic stem and progenitor cell (HSPC) regeneration will be a valuable and a rapid pathway for identifying an effective drug for the treatment of hematopoietic injury.

Here, we report the optimal dose and schedule for the mitigative effects of rHuTPO (administered after exposure) in mice following severe whole body irradiation. The injection of rHuTPO at 25 μg/kg over 14 consecutive days following exposure significantly improved the survival rate of lethally irradiated mice. The administration of rHuTPO notably promoted hematological recovery, increased self-renewing HSPC numbers and enhanced HSPC engraftment in mice following severe total body irradiation (TBI). Our observations indicate that rHuTPO might have a therapeutic role in promoting HSPC regeneration and hematopoietic reconstitution.

## Results

### RHuTPO improves the survival rate of lethally irradiated mice

**Figure 1:**
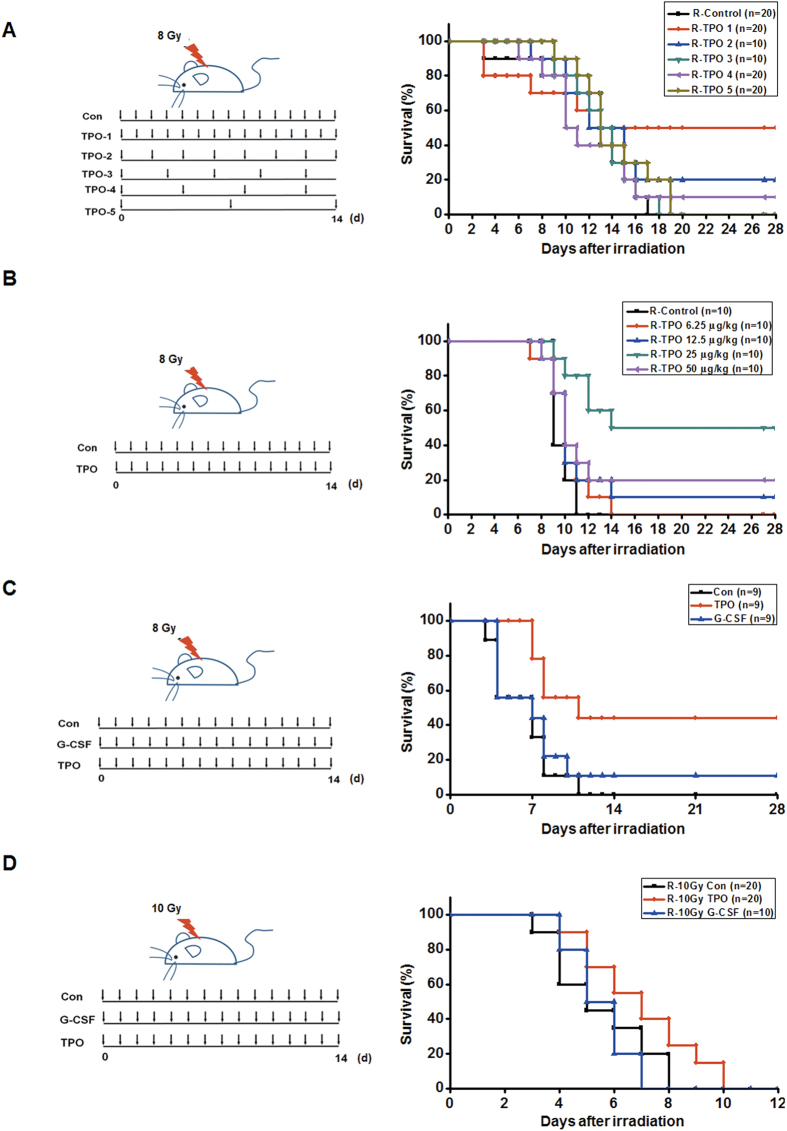
Improved survival following TBI with rHuTPO treatment. (**A**,**B**) Survival curves of the C57BL/6 mice that were irradiated with 8 Gy TBI and received subsequent rHuTPO or PBS treatments for 14 days at different intervals and various doses of rHuTPO. (**C**,**D**) Survival curves of C57BL/6 mice that were irradiated with 8 or 10 Gy TBI and subsequently received rHuTPO, G-CSF or PBS treatments for 14 days. The figure of the mouse was drawn by Dr. Bowen Zhang.

We initially selected the survival rate criteria to evaluate the effectiveness of rHuTPO in alleviating injury to the hematopoietic system and other tissues following radiation. The rHuTPO treatment appeared to be highly effective in improving the survival rate of lethally irradiated mice. To determine an effective schedule of rHuTPO injection following irradiation, mice were subcutaneously administered 25 μg/kg of rHuTPO on time schedules (i.e., injections every one, two, four or seven days for 14 days after radiation). For the injection intervals of one, two and four days, the survival rate of the mice were 50%, 30% and 10%, respectively. In contrast, all of the irradiated mice that received PBS injections died within 17 days (Fig. 1A), which indicated that consecutive injections of rHuTPO were effective in elevating the survival rates of the mice following severe whole body irradiation. To further determine the dose-response relationship of rHuTPO treatment with the improvements in survival rate following lethal irradiation, mice were injected with 0, 12.5, 25, 50 or 100 μg/kg rHuTPO. The 25 μg/kg rHuTPO/day schedule was observed to be the optimal dose and resulted in a 60% survival rate of the mice following severe total body irradiation (Fig. 1B). The improvement in the survival of the mice due to rHuTPO was much better than that of G-CSF (Fig. 1C,D), which indicates that rHuTPO might effectively mitigate the consequences of radiation.

**Figure 2:**
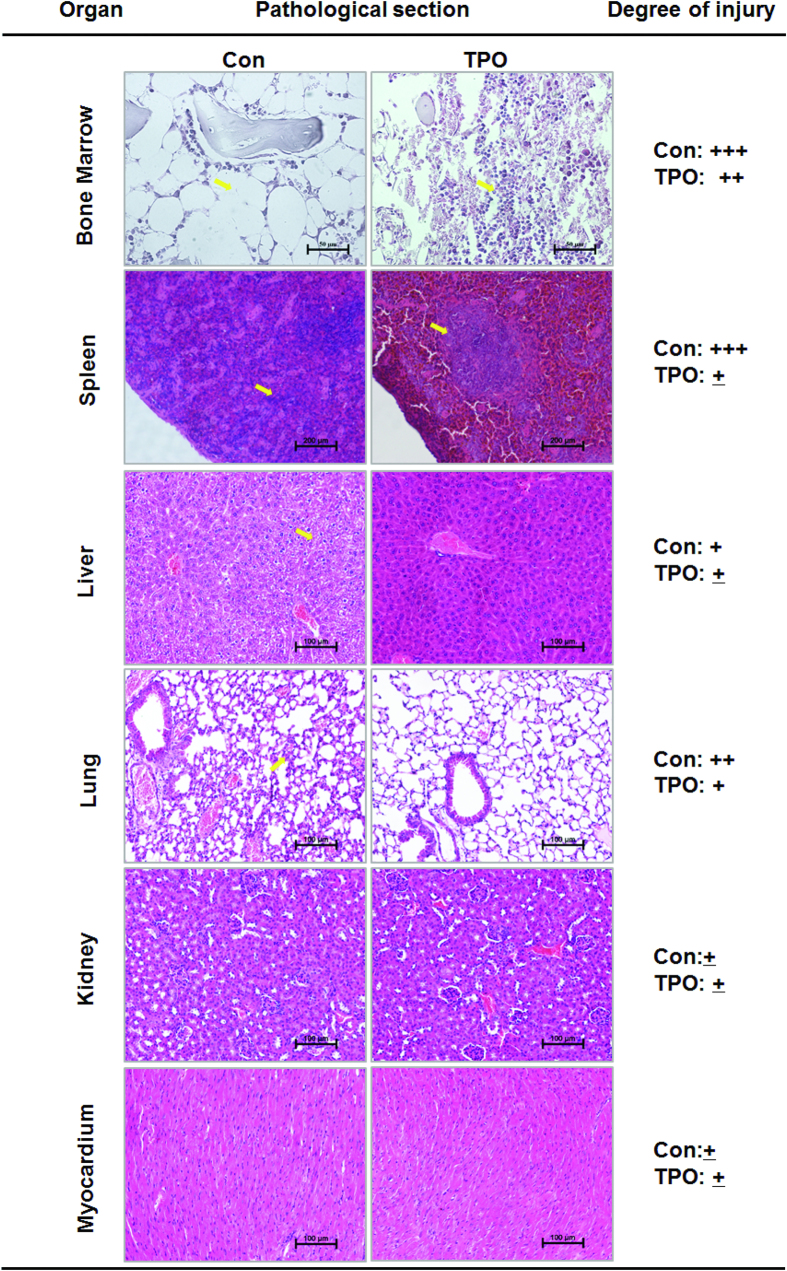
RHuTPO treatment promoted the repair of bone marrow and spleen tissue after TBI. Hematoxylin-eosin staining of the BM, spleen, liver, lung, kidney and myocardium of the rHuTPO-and PBS-treated mice at day 14 after 8 Gy TBI.

On day 14 after irradiation, multiple organs (i.e., the lung, kidney, heart and spleen) were removed from the control and rHuTPO-treated groups and analyzed for histopathologic changes (Fig. 2). No significant pathological changes were observed in the heart or kidney between the two groups. Interestingly, rHuTPO effectively decreased the degree of pulmonary injury and showed a therapeutic effect on interstitial pneumonia caused by irradiation. In addition, rHuTPO treatment significantly decreased hepatic edema, indicating the role of it in enhancing liver tissue repair (Fig. 2). The spleen of control mice showed extensive atrophy of splenic bodies (yellow arrow). In comparison, the spleen from rHuTPO-treated mice showed less atrophy on day 14. Histological analysis of femurs showed that rHuTPO administration remarkably increased bone marrow (BM) cellularity compared to the control group, suggesting that rHuTPO promoted recovery of hematopoietic system.

### RHuTPO promotes hematological recovery

**Figure 3:**
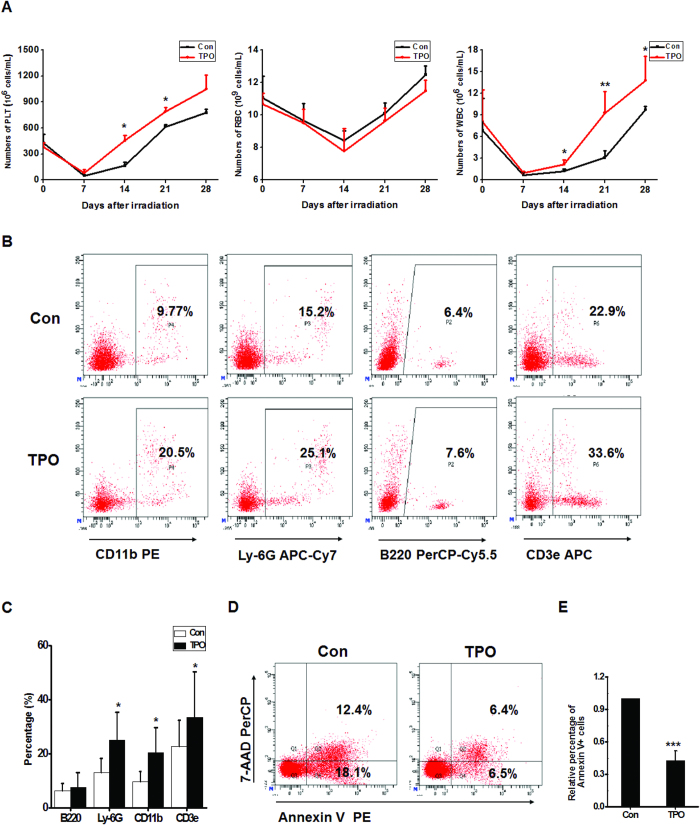
RHuTPO treatment promoted hematological recovery *in vivo* following TBI. (**A**) PLT, WBC and RBC numbers in the PB were calculated at day 14 after TBI. (**B**,**C**) The percentages of myeloid cells and lymphocytes in PB at day 14 after TBI were determined by flow cytometry. (**D**,**E**) Cell death and apoptosis in PB was measured by flow cytometry.

To further investigate the role of rHuTPO in hematopoietic recovery, we first detected hemogram changes. The results suggested that rHuTPO significantly increased peripheral blood (PB) WBC and platelet counts from days 14 to 28 following irradiation (Fig. 3A). Compared with the PBS-injected mice, the percentages of myeloid cells and T lymphocytes were significantly increased in the PB of the rHuTPO-treated mice on day 14 after irradiation (Fig. 3B,C). The results of flow cytometric analyses revealed that the percentage of apoptotic cells in the PB from the rHuTPO-administered mice were notably lower than those in the control group (Fig. 3D,E). These results indicated that rHuTPO might inhibit apoptosis and promote HSPC regeneration after irradiation.

### RHuTPO enhances HSPC regeneration

**Figure 4:**
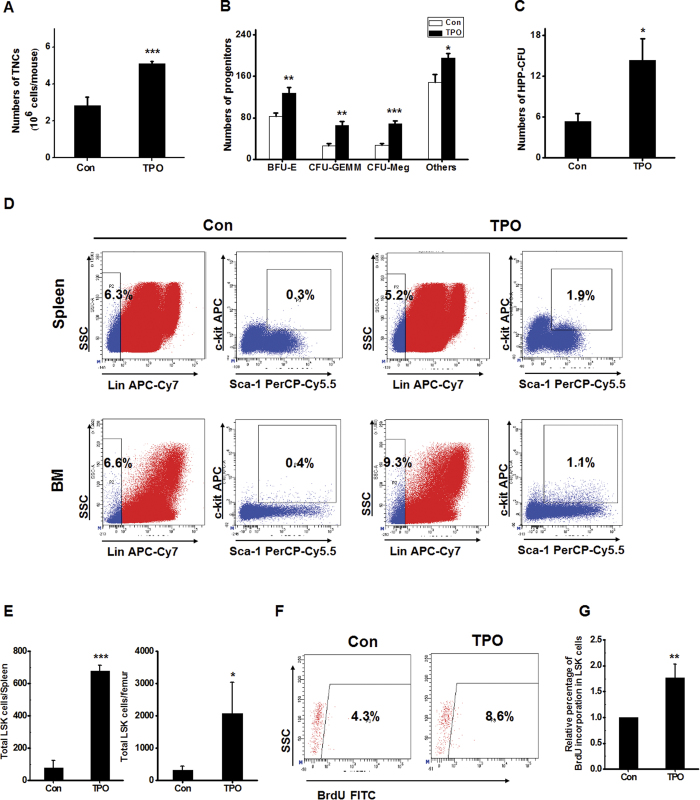
RHuTPO enhanced HSPC regeneration after TBI. (**A**) The numbers of TNCs were calculated from two hind limbs of the rHuTPO-and PBS-treated mice at day 14. (**B**,**C**) Quantification of BM hematopoietic progenitors in the rHuTPO- and PBS-treated mice at day 14 using CFU and HPP-CFU assays. (**D**,**E**) The percentages of LSK cells in the bone marrow and spleen were measured. (**F**,**G**) The BrdU incorporation frequency in the BM LSK cells was measured using flow cytometry.

To investigate whether rHuTPO promotes HSPC regeneration after irradiation, C57BL/6 mice were subjected to 650 cGy TBI and daily subcutaneously injections of rHuTPO for 14 days. Bone marrow cellularity was significantly increased by rHuTPO treatment (Fig. 4A). The mice treated with rHuTPO also exhibited remarkably increased total CFU numbers that included different types of CFUs and HPP-CFUs from the bone marrow (Fig. 4B,C). The percentages of LSK cells in the bone marrow and spleen were much higher in the rHuTPO-treated group than in the control group (Fig. 4D,E), which indicated a role of rHuTPO in promoting HSPC regeneration. BrdU-labeling experiments revealed that the LSK cell populations from the rHuTPO-treated group contained more BrdU^+^ cells than did those of the control mice (Fig. 4F,G), and the percentage of BrdU^+^ cells in LSK CD135^−^ cells were also remarkable increased in mice receiving rHuTPO treatment (Fig. S1A, S1B), which further suggested that rHuTPO enhanced HSPC proliferation after irradiation.

**Figure 5:**
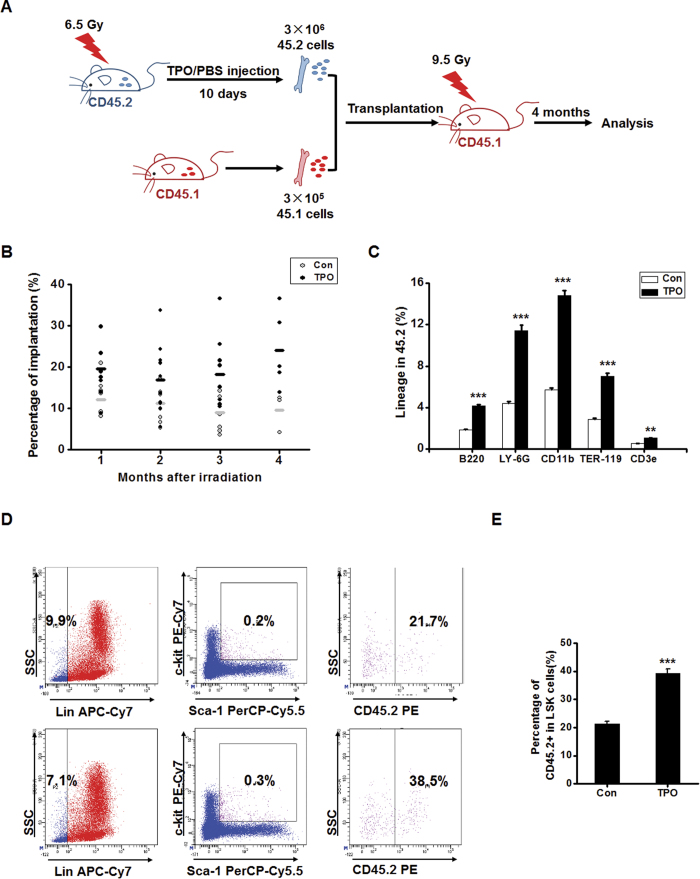
RHuTPO enhanced the repopulating ability of bone marrow hematopoietic cells in a competitive repopulation experiment. (**A**) Schematic of the experimental procedure. The figure of the mouse was drawn by Dr. Bowen Zhang. (**B**) The percentage of CD45.2 donor–derived chimerism was determined by flow cytometry. (**C**) The donor (CD45.2) leukocyte lineages in the PB were analyzed by flow cytometry. (**D**,**E)** The donor (CD45.2) LSK cells in bone marrow at 4 months after transplantation were measured by flow cytometry.

We then performed a competitive repopulation experiment to further assess the repopulating ability of bone marrow hematopoietic cells from rHuTPO-treated mice. C57BL/6 CD45.2 donor BM cells from rHuTPO or PBS-treated mice (3 × 10^6^) in combination with CD45.1 BM cells (3 × 10^5^) were transplanted into lethally irradiated CD45.1 recipients (Fig. 5A). The percentage of CD45.2 donor–derived chimerism was much higher in the recipient mice that received cells from the rHuTPO-treated donor mice (Fig. 5B). Four months after transplantation, the recipient mice were sacrificed, and the PBs were analyzed for the presence of donor (CD45.2^+^) leukocyte lineages. The mice that had been transplanted with bone marrow cells from the rHuTPO-treated mice exhibited more CD45.2-derived B220-, CD3-, CD11b-, Ly-6G- and Ter-119- positive cells than did the mice that received cells from the control mice (Fig. 5C). Correspondingly, the donor LSK cell frequency in the bone marrow of recipients receiving cells from the rHuTPO-treated mice was much higher than that of the recipients with engrafted cells from the PBS-treated mice (Fig. 5D,E). These results strongly indicated that rHuTPO increased the numbers of HSPC with multilineage differentiation potential in mice after TBI.

### RHuTPO promotes HSPC engraftment

**Figure 6:**
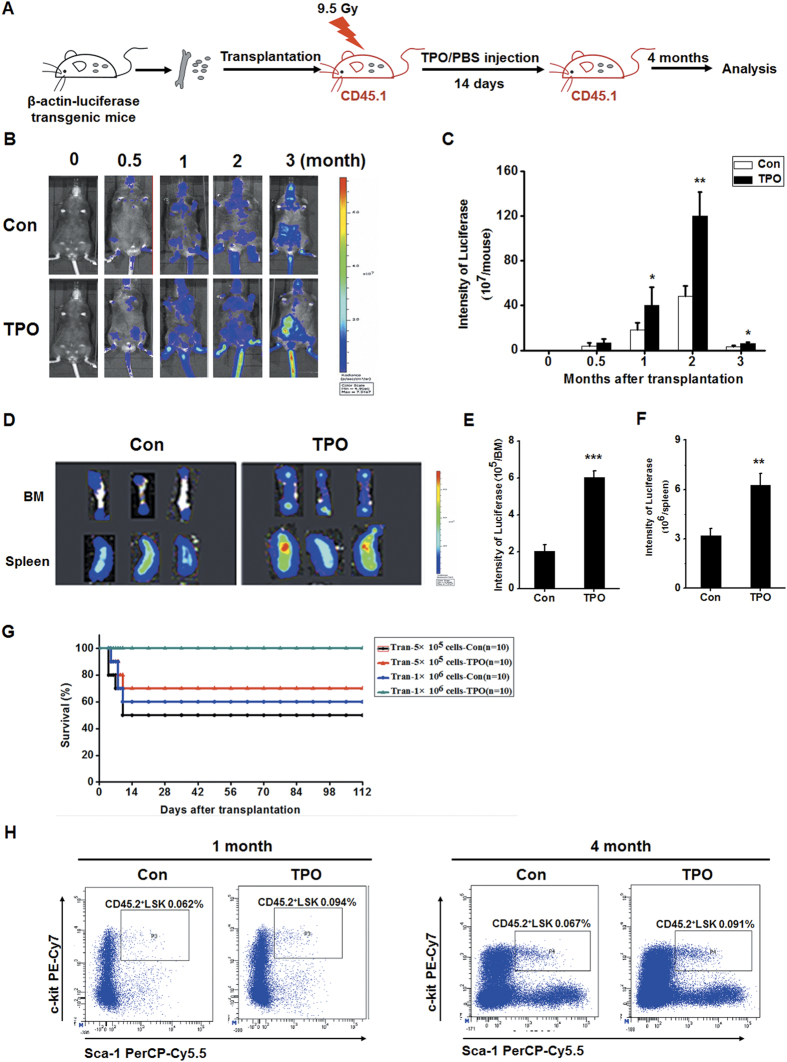
RHuTPO promoted HSPC engraftment after TBI. (**A**) Schematic of the experimental procedure. The figure of the mouse was drawn by Dr. Bowen Zhang. (**B**,**C**) Donor bone marrow cell engraftment was determined by *in vivo* fluorescence imaging. (**D–F**) Donor bone marrow cell engraftment in the BM and spleen was determined by *in vivo* bioluminescence imaging. (**G**) Survival curves of the C57BL/6 mice that were given 9.5Gy TBI followed by the transplantation of normal bone marrow cells and rHuTPO or PBS treatments for 14 days. (**H**) The donor LSK cell frequency in the recipient bone marrow four months after TBI and BM transplantation was measured by flow cytometry.

To further investigate the role of rHuTPO in enhancing HSPC engraftment, lethally irradiated CD45.1 mice were transplanted with bone marrow cells from β-actin-luciferase transgenic mice and received rHuTPO or PBS treatment for 14 consecutive days (Fig. 6A). The *in vivo* bioluminescence imaging results revealed that the donor bone marrow cells were more thoroughly engrafted in the rHuTPO-treated recipient mice than in the PBS-treated mice, particularly at 0.5–3 months after transplantation (Fig. 6B,C). Importantly, rHuTPO treatment significantly increased transplanted hematopoietic cell engraftment in the bone marrow and spleen of the recipients (Fig. 6D–F). Given that hematopoietic cytokines exhibited little effect on BM reconstruction in the mice after lethal irradiation, we transplanted normal bone marrow cells into the recipient mice after 9.5 Gy exposure and 14 consecutive days of injections of rHuTPO or PBS. We found that 100% of the mice that received 1 × 10^6^ BM cells and 14 consecutive days of rHuTPO treatment survived. In contrast, 60% of the recipients in the control treatment group survived. The transplantation of 5 × 10^5^ bone marrow cells rescued 70% of the recipient mice that received rHuTPO treatment. A lower survival rate was observed in the mice that received bone marrow cells and PBS treatment (Fig. 6G). These results suggested that rHuTPO enhanced the survival of lethally irradiated mice following bone marrow transplantation. The rHuTPO treatment group also exhibited a significantly increased frequency of donor CD45.2^+^ LSK cells in the bone marrow (Fig. 6H). These data suggested that rHuTPO enhanced HSPC engraftment in lethally irradiated mice.

## Discussion

The hematopoietic system is the most vulnerable target of radiation injury. Hematopoietic reconstitution is critical for rescuing patients from the effects of chemotherapy or radiotherapy. Modulating HSPCs, which are the key seed cells for hematopoietic reconstitution, requires both extrinsic and intrinsic factors. Several hematopoietic cytokines have been clinically used to alleviate the symptoms of neutropenia, anemia and thrombocytopenia. The development of new treatments is critical for effectively reconstituting the hematopoietic system following radiation-induced injury.

TPO was first cloned and its recombinant protein first produced in 1994^22^. Two recombinant forms of the protein, full-length recombinant human TPO and pegylated recombinant human megakaryocyte growth and development factor (PEG-rHuMGDF) were then evaluated as a regulator of megakaryopoiesis and platelet production in patients^34^. However, PEG-rHuMGDF ended to be used in clinic due to the production of antibodies to it^35^. Indeed, TPO is more than a lineage-specific megakaryocyte growth factor. It also exerts a remarkable influence on HSPCs especially promoting hematopoiesis and hematopoietic progenitor cell expansion^36^. However, the clinical application of rHuTPO, approved by the State Food and Drug Administration (SFDA) of China in 2005, is still limited to immune thrombocytopenia and chemotherapy-related thrombocytopenia^31^,^32^,^33^. The second-generation TPO receptor agonists also showed preliminary success in the treatment of thrombocytopenia^37^. Based on the effect of TPO with its receptor on HSPC proliferation, we predicted that the administration of rHuTPO to recipients would enhance hematopoietic reconstitution. In the initial stage of the study, we first evaluated the influence of rHuTPO on the survival rate of irradiated mice, which might reflect the degree of hematopoietic recovery, and the optimal schedule of rHuTPO administration. The administration of rHuTPO to recipients revealed its capacity to improve the survival rate of lethally irradiated mice with or without bone marrow transplantation. Indeed, the injection of 25 μg/kg rHuTPO for 14 consecutive days post-exposure exhibited a much better effect than the other dose schedules, which indicated that the maintenance of a stable concentration of rHuTPO was required for hematopoietic cell regeneration. Daily rHuTPO treatment following irradiation remarkably enhanced the HSPC numbers in the bone marrow and spleen and further elicited rapid recoveries of WBCs and platelets. These results indicated that rHuTPO promoted residual HSPC proliferation. The enhanced BrdU incorporation in response to rHuTPO administration also reflected the ability of rHuTPO to stimulate HSPC regeneration *in vivo*. More importantly, the results of the competitive reconstitution experiment further indicated that rHuTPO administration increased the numbers of long-term HSCs. These results provide important evidence for the role of rHuTPO in the regulation of HSPC self-renewal and indicate that rHuTPO might initiate HSPC renewal and proliferation in patients after chemotherapy or radiotherapy. A recent study suggested that TPO administration prior to irradiation limits HSPC mutagenesis by increasing DNA-PK-dependent DNA repair^38^. RHuTPO treatment before and after radiotherapy might produce much better effects on hematopoietic system reconstitution than administration only after radiation.

In the clinic, myeloablative therapy and HSPC transplantation are often used in patients with malignant or non-malignant hematological diseases. RHuTPO is used when a patient exhibits symptoms of thrombopenia. Until now, no drug has been used effectively to enhance HSPC engraftment. Here, we found that the administration of rHuTPO promoted hematopoietic cell engraftment and increased the survival rate of lethally irradiated mice following bone marrow transplantation. The recipients that received 5 × 10^5^ bone marrow cells and the daily rHuTPO treatment exhibited a survival rate that was comparable to that of the mice that received 1 × 10^6^ bone marrow cells and placebo injection. This result suggested that rHuTPO might be effective in improving the limited efficiency of hematopoietic reconstitution that follows limited HSPC transplantations, such as cord blood transplantation or limited peripheral blood stem cell transplantation, due to a poor HSPC mobilizing effect. The injection of 25 μg/kg rHuTPO for 14 consecutive days resulted in much greater numbers of donor LSK cells in the recipients with BMT, which indicated that rHuTPO enhanced long-term hematopoietic repopulation. There are some discrepancies between our results and those of other reports^39^,^40^ which might be due to the different doses, schedules and drug sources used. A recent study suggested that HSC subtypes can be organized into a cellular hierarchy with platelet-primed HSCs at the apex^41^. TPO is crucially required for the maintenance of platelet-primed HSCs. This opinion further supports the administration of rHuTPO to patients following HSPC transplantation, which might promote platelet-primed HSPC self-renewal and proliferation and speed up platelet recovery.

## Conclusion

Together, the results of this study provide strong evidence supporting the therapeutic application of rHuTPO for hematopoietic reconstitution. The administration of rHuTPO after radiation exposure can stimulate HSPC proliferation, promote HSPC engraftment and improve the survival rate of mice. These data further support the application of rHuTPO for patients undergoing radiotherapy or following HSPC transplantation. The therapeutic value of rHuTPO in patients requires further evaluation in primate models and clinical trials.

## Methods

### Mice, radiation and treatment

Adult CD45.1 and CD45.2 C57BL/6 mice (eight weeks old, male, 20–25 g) were purchased from the China Academy of Medical Sciences Animal Center (Beijing, China). Luciferase-reporter transgenic CD45.2 C57BL/6 mice were purchased from Keyuandi Biotech Co. (Shanghai, China). All animal experiments were reviewed and approved by the animal center committee of the Academy of Military Medical Sciences (Beijing, China), and the animals were housed and handled in accordance with the guidelines of the National Institutes of Health.

Total body irradiation (TBI) was administered at day 0 using a Co^60^ irradiator at a dose of 1400 to 1500 cGy/min. Eight-gray doses were used in the survival experiment. For evaluation of the radiation-mitigating efficacy of rHuTPO, mice were irradiated at 6.5 Gy. For hematopoietic repopulation experiments, the recipient mice were irradiated at 9.5 Gy (twice, 4.5 Gy and 5 Gy with 1 hour intervals).

The rHuTPO was produced in Chinese hamster ovary cells (Shenyang Sunshine Pharmaceutical Co., China) and diluted in phosphate-buffered saline/0.01% BSA. Irradiated mice were injected with rHuTPO or PBS (control) daily for 14 days. In survival experiments, rHuTPO or PBS will be injected for different intervals that included once per day, once every two days, once every three days and once every week.

### Analysis of white blood cells

Peripheral blood cells were obtained from irradiated mice with different treatment. White blood cell (WBC) counts, including lymphocytes and neutrophils, were enumerated using a Sysmex Microcell Counter.

### Colony assays

Bone marrow cells were isolated and CFU assays were performed as previously described^42^. Briefly, bone marrow mononuclear cells (MNCs) were collected and cultured in colony culture medium. Seven days later, typical colonies, including granulocyte-erythroblast-macrophage-megakaryocyte colony-forming units (CFU-GEMM), erythrocyte burst-forming units (BFU-E), granulocyte-macrophage colony-forming units (CFU-GM), megakaryocyte colony-forming units (CFU-Meg) and granulocyte colony-forming units (CFU-G), were visually scored based on morphologic criteria using a light microscope, and the total numbers of CFUs were calculated. The cultures were incubated for 10–14 days, and the numbers of high proliferative potential colony-forming units (HPP-CFU) were counted.

### Flow cytometry analysis

BM cells were flushed from the femurs of mice with PBS containing 2% FBS. BM RBCs were lysed with lysing buffer (BD). For blood cell lineage detection, the cells were stained with lineage antibodies against CD3, CD11b, Ter-119, B220, Ly-6G and matched isotype controls (eBioscience). To examine the percentages of LSK cells, bone marrow cells were suspended in PBS and incubated with APC-Cy7-labeled lineage antibodies, PerCP-Cy5.5-conjugated anti-Sca-1 and APC-conjugated anti-c-Kit antibodies for 30 min. For the detection of engraftment and chimerism, the bone marrow cells were stained with PE or FITC-labeled antibodies against CD45.1 or CD45.2 (eBioscience). To monitor the proliferating hematopoietic cells, the mice were intraperitoneally injected with BrdU (100 mg/kg body weight) 12 h before sacrifice. The BrdU incorporation assay was performed using a cell proliferation assay kit (Sigma). Cell apoptosis was measured using a PI and Annexin-V staining kit according to the instructions of the manufacturer.

### Transplantation experiments

For competitive transplantation experiments, bone marrow MNCs isolated from irradiated CD45.2 mice treated with rHuTPO or PBS for 10 days. Both 3 × 10^6^ MNCs (CD45.2) and 3 × 10^5^ congenic BM competitor cells (CD45.1) were transplanted into lethally irradiated (950 cGy) CD45.1 mice. At every month after transplantation, the numbers of CD45.1^+^ and CD45.2^+^ cells were detected by flow cytometry. At 4 months after transplantation, the multilineage reconstitution capacity of donor cells was analyzed by flow cytometry.

For noncompetitive transplantation experiments, mice were subjected to lethally whole-body irradiation (800 cGy) and then transplanted with 5 × 10^5^ or 1 × 10^6^ bone marrow MNCs isolated from C57BL/6 donor mice. The recipient mice were administered with rHuTPO (25 μg/kg) or PBS (control) daily for 14 days. The survival rates of the mice were monitored for 4 months. The reconstitution capacity of donor cells was analyzed by flow cytometry. Optical bioluminescence imaging of mice after luciferase-reporter expressing bone marrow cell transplantation was conducted with a CCD camera (Xenogen Corp., Alameda, CA).

### Statistics

Unless otherwise noted, the *P* values were calculated using unpaired 2-tailed Student’s *t* tests, and *P* values below 0.05 were considered significant. All error bar data represent the mean ± the SD.

## Additional Information

**How to cite this article**: Wang, C. *et al.* Recombinant human thrombopoietin promotes hematopoietic reconstruction after severe whole body irradiation. *Sci. Rep.* **5**, 12993; doi: 10.1038/srep12993 (2015).

## Supplementary Material

Supplementary Information
