# Supplementary material for: Recombinant human thrombopoietin promotes hematopoietic reconstruction after severe whole body irradiation
Source: Sci Rep. 2015 Sep 25;5:12993. doi: 10.1038/srep12993 (PMC4585893; doi:10.1038/srep12993)
Supplement: Supplementary Information [file srep12993-s1.pdf]

Supplementary Information

Recombinant human thrombopoietin promotes hematopoietic reconstruction after severe whole body irradiation

Chao Wang[1,3*], Bowen Zhang[1,2*], Sihan Wang[1,2], Jing Zhang[1,2],Yiming Liu[1,2],Jingxue Wang[1,2], Zeng Fan[1,2], Yang Lv[1,2], Xiuyuan Zhang[1,2], Lijuan He[1,2], Lin Chen[1,2], Huanzhang Xia[3**], Yanhua Li[1,2**], Xuetao Pei[1,2**]

[1]Stem Cell and Regenerative Medicine Lab, Beijing Institute of Transfusion Medicine, Beijing 100850, China, [2]South China Research Center for Stem Cell & Regenerative Medicine, AMMS, Guangzhou 510005, China, [3]School of Life Science and Bio Pharmaceutics, Shenyang Pharmaceutical University, Shenyang 110016, China.

[*]These authors made an equal contribution.

[**]Correspondence to Huanzhang Xia (xiahz612@sina.com), Yanhua Li (shirlylyh@126.com) or Xuetao Pei (peixt@nic.bmi.ac.cn).

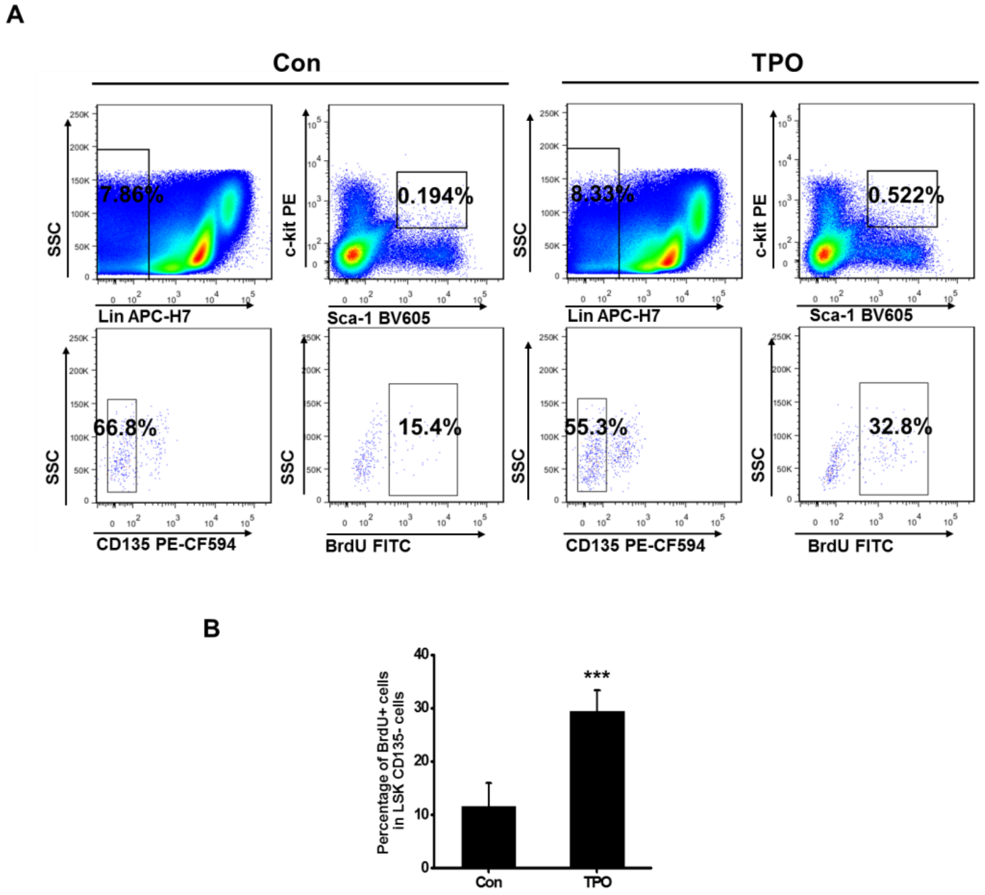

Supplementary Figure Legend

The BrdU incorporation frequency in the BM LSK $CD135^{-}$ cells was measured using flow cytometry.
